# Are there any interactions between modified Nordic-style diet score and MC4R polymorphism on cardiovascular risk factors among overweight and obese women? A cross-sectional study

**DOI:** 10.1186/s12902-022-01132-1

**Published:** 2022-09-01

**Authors:** Dorsa Hosseininasab, Atieh Mirzababaei, Faezeh Abaj, Roya Firoozi, Cain C. T. Clark, Khadijeh Mirzaei

**Affiliations:** 1 Department of Nutrition, Science and Research Branch, Islamic Azad University, Tehran, Iran; 2 Department of Community Nutrition, School of Nutritional Sciences and Dietetics, Tehran University of Medical Sciences (TUMS), P.O. Box:14155-6117, Tehran, Iran; 3 Department of Nutritional Sciences, School of Public Health, University of Michigan, Ann Arbor, MI USA; 4 Centre for Intelligent Healthcare, Coventry University, Coventry, CV1 5FB UK

**Keywords:** Obesity, MC4R polymorphism, Modified Nordic-style diet score

## Abstract

**Background:**

Cardiovascular disease (CVD) is the leading cause of death in women globally. Recent studies have reported that the minor allele (C allele) for melanocortin 4 receptor (MC4R) rs17782313 may be related to the incidence of obesity and the risk of CVD. Therefore, the present study aimed to investigate the interactions between the modified Nordic-style diet score (MND) and MC4R gene variant on markers of CVD.

**Methods:**

The current cross-sectional study was conducted on 282 Iranian women, aged 18–48 years, with a body mass index (BMI) ≥ 25. MND score was assessed using a 147 items food frequency questionnaire (FFQ). Genotyping of the MC4R (rs17782313) was conducted by the PCR method. The anthropometric measurements and serum profiles were assessed by standard protocols.

**Results:**

The means and standard deviation (SD) of age, weight, and BMI of individuals were 36.67 ± 9.10 years, 81.29 ± 12.43 kg, and 31.26 ± 4.29 kg/m^2^, respectively. The overall prevalence of rs17782313 genotypes was 30.1%, 24.8%, and 45.1% for TT, TC, and CC, respectively. Our results showed significant negative interactions between high MND score and rs17782313 SNP in terms of visceral fat level (VFL) (β: -10.84, 95% CI: -20.64 to -1.04, *P* = 0.03) and total cholesterol (β: -24.24, 95% CI: -49.87 to 1.38, *P* = 0.06) in the crude model. After adjusting confounders, the interaction between high MND scores and VFL remained significant.

**Conclusion:**

In conclusion, the results of the present study suggest that diet, gene variants, and their interaction should be considered in metabolic disease risk assessment. Further studies are needed to confirm these data and better elucidate the interaction.

**Supplementary Information:**

The online version contains supplementary material available at 10.1186/s12902-022-01132-1.

## Introduction

Cardiovascular disease (CVD) is a leading cause of death globally, where estimates indicate that more than 82 million American adults (1 in 3) have one or more varieties of CVD [1, 2]. CVD represents the main cause of death in developed and developing countries [3, 4] and its’ incidence is projected to reach 23.6 million worldwide by the end of 2030 [5]. In general, 1 in 3 women die from CVD and 45% of women over the age of 20 have some type of CVD [6, 7], whilst some studies suggest that around 44.8% of deaths in Iran will be due to CVD by 2030 [8]. Some studies have suggested that several factors such as age, gender, total cholesterol, and high-density lipoprotein (HDL) cholesterol concentration might be related to the CVD and mortality [9, 10]. One of the most important risk factors for CVD is obesity.

The association of overweight and obesity with dyslipidemia, hypertension (HTN), and diabetes, which also contribute to the risk of developing CVDs themselves, has also been substantiated [11, 12]. The pathogenesis of obesity is influenced by the balance between calories consumed and energy expenditure [13]. Diet is one of the most influential variables in lifestyle that plays a crucial role in the inhibition of non-communicable diseases, such as CVD, obesity, and their main risk factors [14].

The role of diet in modulating the risk of non-communicable diseases is supported by several studies [15]. Indeed, numerous studies have revealed that better adherence to healthy eating patterns, like the Mediterranean Diet Score (MDS), the Dietary Approaches to Stop Hypertension (DASH), and the Healthy Eating Index (HEI), considerably reduces CVDs [16–18]. Recently, the Nordic diet (ND), a healthy diet based on foods usually consumed in Nordic countries, including Norway, Denmark, Sweden, Iceland and Finland [19], has gained interest from scientists. This diet contains berries, fruits (e.g. apple and pear), legumes, vegetables, whole grains, and dietary fiber from oats, barley, and rye; low-fat dairy; fatty fish, and rapeseed oil [19]. It also includes only a small amount of salt, added sugar, and saturated fats in the diet [19–21]. The ND can be beneficial in preventing CVDs and type 2 diabetes [22–24]. In randomized controlled trials, the ND yielded beneficial effects, similar to the Mediterranean diet, on cardiac metabolic risk factors [25–27].

Genetic factors are also key parameters that can modulate the expression of obesity, as well as CVD [28]. The most prevalent genetic cause of obesity is a mutation in the Melanocortin 4 receptor (MC4R) gene, which encodes the melanocortin 4 receptor [29] and is expressed in the hypothalamus [30, 31], and obesity, indirectly, could increase the risk of death from CVDs [32]. Cardiovascular risk factors, such as HTN, have also been associated with the risk allele C for MC4R rs17782313 [33]. Inactivated MC4R gene is associated with lower blood pressure (BP), independently of obesity [34]. Nevertheless, this association has been displayed only in individuals with mutations [34]; for instance, Khodarahmi et al., reported that MC4R rs17782313 interacts with healthy dietary patterns (DASH score and MDS) to influence cardiometabolic risk factors and hypothalamic hormones in obese individuals [35]. Another study showed that dietary quality indices modified the effects of MC4R rs17782313 polymorphism on cardiometabolic risk factors and hypothalamic hormones in obese males and females [36]. The reason why we chose ND was that several studies have shown a positive association between adherence to the ND with obesity traits and the risk of non-communicable chronic diseases such as CVDs and also the risk of mortality in the Iranian population [37–40].

Since those with polymorphisms near the MC4R gene are more prone to suffer from obesity [41–43], obesity contributes directly to incidence of cardiovascular risk factors [44, 45], and since there is purportedly a positive relationship between adherence to ND and anthropometric measurements in recent studies, we sought to assess the interaction between MND and MC4R polymorphism on cardiovascular risk factors among overweight and obese Iranian women.

## Methods

### Study participants

The current cross-sectional study was performed on 282 women who were referred to health centers, selected by multistage random sampling in Tehran, Iran. Inclusion criteria were volunteers aged 18 to 48 years, overweight or obese (overweight: body mass index (BMI) 25–29.9 kg/m^2^, obesity: BMI ≥ 30 kg/m^2^), and agreed to participate in the study. The exclusion criteria were presence of acute or chronic infection, history of type 2 diabetes, CVD, dyslipidemia, polycystic ovary syndrome (PCOS), stroke, non-alcoholic fatty liver disease (NAFLD), inflammatory diseases, HTN, cancer, and thyroid disease, taking drugs that could create weight changes or affect BP, blood lipoproteins, blood glucose, supplementation (multivitamins) or alcohol consumption, pregnancy, lactation, or menopause, as well as special energy consumption outside the range of 800–4200 kcal/day [46]. This study protocol was approved by the ethics committee of the Tehran University of Medical Sciences (IR.TUMS.VCR.REC.1398.619), and written informed consent was signed by all participants before taking part in the study.

### Dietary assessment and calculation of modified Nordic-style diet score

The regular dietary intake of participants was calculated using a validated semi-quantitative food frequency questionnaire (FFQ) containing 147 food items [47]. Participants were asked to report their consumption frequency of one other food item per day, week, month, or year. Food intakes reported in household sizes were then transmuted to grams of food per day utilizing the nutritionist IV software [48]. Nutrient intakes were computed by Nutritionist IV software, which was modified for Iranian foods based on the United States Department of Agriculture (USDA) food composition table [49]. The FFQ was completed by a trained nutritionist. We computed the median consumption of the food groups according to the FFQ. Intakes above and below the median were given 1 and 0 points, respectively. The score of each group was summed and classified: 0–1 point for low adherence, 2–3 points for medium adherence, and 4–6 points for high adherence. Since our population did not consume an adequate quantity of foods listed in the main Nordic-style diet score [50], we used a modified Nordic-style diet score, comparable to another study which was conducted on an Iranian population [39] by utilizing data from 6 food groups with similar micronutrient amounts containing; (1) rye and wholegrain bread with a median of 90, (2) oatmeal (chickpea, bean, lentil, oat, soybean, frumenty, split pea, mung bean, and vicia faba) with a median of 20, (3) vegetables and cabbages (cucumber, celery, lettuce, tomato, raw and boiled spinach, zucchini, bell pepper, and leafy vegetables) with a median of 132, (4) pears, apples, and high antioxidant fruits (peach, apricot, dry apricot, pear, apple, apple juice, nectarine, strawberry, persimmon, mulberries, dry mulberries, plum, and dry plum) with a median of 97, (5) root vegetables (raw and boiled carrot, potato, onion, garlic, and turnip) with a median of 41 and (6) fish (fish conserved in oil and salt and other fish) with a median of 2 [39, 51, 52].

### DNA extraction

The MC4R gene primer was chosen based on a previous study [53]. Following the manufacturer’s protocol, we extracted genomic DNA from blood samples using the Mini Columns, Type G kit (GeneALL, Exgene). Moreover, with the use of a NanoDrop spectrophotometer (Thermo Scientific Company, USA), we assessed the concentration and purity of extracted DNA. We stored the extracted DNA at 4ºC before sequencing was carried out. The polymerase chain reaction (PCR) was carried out utilizing the following primers: forward primer 5 AAGTTCTACCTACCATGTTCTTGG-3 and reverse primer 5- TTCCCCCTGAAGCTTTTCTTGTCATTTTGAT-3. PCR reactions were performed in a final volume of 20 μl, containing 1 μl extracted DNA, 0.5 μl primers F, 0.5 μl primers R, 10 μl Permix (Amplicon, Germany), and 8 μl distilled water, with the following conditions in a DNA thermocycler: 1- primary denaturation at 95 °C for 2 min; 2- Thirty- five cycles of denaturation at 95 °C for 30 s, annealing at 58 °C for 30 s, extension at 72 °C for 30 s; 3- final extension at 72 °C for 5 min; 4- final step at 4 °C. 0.5 μl of BCII restriction enzyme (Fermentase, Germany) was used to digest amplified DNA (7 μl) at 56 °C overnight. All products were visualized by agarose gel electrophoresis [54]. After that, fragments containing three genotypes were distinguished: CC, CT, and TT.

### Assessment of anthropometric measurements

Participants' height was evaluated with a Seca stadiometer with an accuracy of nearly 0.1 cm. Weight was measured utilizing a digital scale (Seca, Hamburg, Germany) in thin clothing without shoes with an accuracy of nearly 0.1 kg. Waist circumference (WC) was assessed at the smallest distance between the lower end of the sternum (xiphoid process) and the umbilicus [55] and hip circumference (HC) was measured at the widest region of the hip [56]. The mean of the two closest readings was used in the statistical analysis. Waist-to-hip ratio (WHR) was also calculated. All measurements were performed by an expert nutritionist and followed the standard protocols [57]. Participants were assessed for anthropometric indices in the Nutrition and Biochemistry Laboratory of the School of Nutrition and Dietetics, TUMS.

### Assessment of body composition

Body composition parameters, including amount and proportion of body fat mass (BFM), fat-free mass (FFM), and visceral fat level (VFL), were taken by multi-frequency bioelectrical impedance analyzer (BIA): InBody 770 Scanner (InBody Co., Seoul, Korea). Measurements were performed in the morning in a fasted condition with light clothing. Participants were asked not to exercise, not to carry any electric devices, and to urinate just before the body composition analysis, to yield a more accurate result. According to manufacturer instructions, participants stood on the scale with bare feet and held the handles of the machine for 20 s, then the output was printed. The precise measurement method has been described in detail elsewhere [58].

### Biochemical parameters

All participants' blood samples were collected in the morning, after a 10–12 h fasting. Fasting blood sugar (FBS) and triglyceride (TG) were evaluated by the Glycerol-3phosphate oxidase Phenol 4-Aminoantipyrine Peroxidase (GPO-PAP) method. Total cholesterol was evaluated by the cholesterol oxidase Phenol 4-Aminoantipyrine Peroxidase (CHOD-PAP). The direct method and immunoinhibition assay were used to measure low-density lipoprotein (LDL) and HDL. Serum insulin values were assessed by radioimmune assay. The insulin resistance homeostatic model assessment (HOMA-IR) was computed: [fasting plasma glucose (mmol/l) × fasting plasma insulin (mIU/l)]/22.5. For quantifying serum high sensitivity C-reactive protein (hs-CRP) levels, an immunoturbidimetric test was used. All assessments kits were from Pars Azmoon (Pars Azmoon Inc. Tehran, Iran).

### Assessment of blood pressure

Participants' BP was measured twice (with an interval of 2 min) on the left arm by a trained physician, after 15 min of sitting, using a standard sphygmomanometer (Omron, Germany, Europe). Their mean was calculated and recorded as the BP.

### Assessment of other variables

Demographic characteristics, including age, level of education, marital status, specific diet, medical history, medication, and supplementation, were completed by a trained nutritionist. Physical activity (PA) was evaluated by a validated international physical activity questionnaire (IPAQ), which was computed as metabolic equivalent hours per week (METs h/week) [59]. Participants were asked by trained interviewers to report about vigorous and moderate activities pertaining to the last 7 days. To compute the activity, the length and frequency of activity days were multiplied. The sum of the grades was computed as the total exercise per week and PA was classified as: low (< 600 METs h/week), moderate (600–3000 METs h/week), and severe (> 3000 METs h/week) [60].

### Statistical analysis

Genotype groups of the MC4R were considered as a dominant inherent model (TC + CC) versus TT homozygous. They were recoded based on risk allele: code 0 for TT and 1 for TC + CC. The Kolmogorov–Smirnov test was used for assessing the normality of the data. The Hardy–Weinberg equilibrium and comparison of categorical variables were assessed with the Chi-square test. Comparison of quantitative variables between tertiles of MND or MC4R genotypes (TT vs. TC + CC) was performed using independent samples T-tests and analysis of covariance (ANCOVA). The interaction between MND and MC4R genotypes on quantitative variables was assessed using generalized linear regression model (GLZM) analysis. All statistical analysis were performed using SPSS v25 software. Accordingly, *P* < 0.05 was considered statistically significant, but for interactions, *P* < 0.1 was considered significant.

## Results

### Study population characteristics

The current cross-sectional study was conducted on 282 women classified as overweight or obese. 44.1% and 55.9% of the participants were overweight and obese, respectively. Also, 97% of the individuals had high body fat. The means and standard deviations (SD) of age, weight, and BMI of individuals were 36.67 ± 9.10 years, 81.29 ± 12.43 kg, and 31.26 ± 4.29 kg/m^2^, respectively. 6.9%, 21.7%, 21.3%, 63.5%, and 8.2% of participants had high levels of LDL, TG, cholesterol, HOMA-IR, and FBS, and 35.2% had low levels of HDL (normal ranges are presented in supplementary table 1). The overall prevalence of rs17782313 genotypes was 30.1%, 24.8%, and 45.1% for TT, TC, and CC, respectively.

### Association between biochemical parameters, body composition and MND

All participants were divided into three groups, based on the MND score (low intake, moderate intake, high intake). Before adjustment for BMI, age, total energy intake, and PA, significant differences between groups were found for fat-free mass (FFM) (*P* = 0.03); in other words, participants who had higher adherence to MND had higher levels of FFM. After adjustment for BMI, age, total energy intake, and PA, higher quartiles of MND were associated with lower levels of VFL (*P* = 0.04) (Table 1).

**Table 1:** Characteristics of the study participants among MND tertiles

**Variable**	**T1** (*n* = 73)	**T2** (*n* = 160)	**T3** (*n* = 169)	***P*-value**	*P*-value*
**Demographic variables**					
Age (years)	36.42 ± 9.51	35.85 ± 9.10	37.54 ± 8.89	0.23	0.23^b^
PA (MET-minutes/week)	989.46 ± 1925.63	1031.06 ± 1994.74	1409.05 ± 2200.22	0.32	0.29^b^
**Anthropometric parameters**					
Weight (kg)	78.40 ± 11.17	81.52 ± 12.86	82.32 ± 12.41	0.07	0.06^a^
Height (m)	160.10 ± 5.70	161.56 ± 5.87	161.38 ± 5.90	0.18	0.15^a^
BMI (kg/m^2^)	30.81 ± 4.58	31.16 ± 4.40	31.54 ± 4.06	0.45	0.58^b^
WC (cm)	98.20 ± 9.59	99.62 ± 10.56	100.19 ± 9.80	0.37	0.74^a^
WHR	0.93 ± 0.04	1.50 ± 7.19	0.93 ± 0.05	0.47	0.77^a^
**Body composition**					
BFM (kg)	33.71 ± 8.45	34.96 ± 9.27	34.96 ± 8.38	0.55	0.72^a^
FFM (kg)	45.20 ± 4.75	46.36 ± 5.79	47.25 ± 5.93	**0.03**	0.44^a^
VFL	18.31 ± 22.92	15.96 ± 3.35	16.79 ± 10.76	0.39	**0.04**^**a**^
**Blood pressure**					
SBP (mmHg)	109.46 ± 12.46	109.95 ± 15.58	113.28 ± 14.86	0.13	0.30
DBP (mmHg)	78.86 ± 10.32	77.25 ± 10.29	77.42 ± 10.56	0.64	0.77
**Blood parameters**					
FBS (mmol/dl)	87.45 ± 11.26	87.36 ± 9.84	87.60 ± 8.99	0.98	0.81
HOMA-IR	3.33 ± 1.18	3.41 ± 1.36	3.31 ± 1.23	0.85	0.89
Total Cholesterol (mg/dl)	190.62 ± 36.95	180.88 ± 35.46	186.91 ± 35.53	0.28	0.52
TG (g/dl)	106.50 ± 47.84	121.10 ± 65.88	127.94 ± 76.91	0.23	0.29
HDL (mg/dl)	46.57 ± 10.05	46.32 ± 9.83	46.78 ± 11.88	0.95	0.64
LDL (mg/dl)	91.45 ± 22.22	95.33 ± 24.25	96.52 ± 24.67	0.51	0.54
LDL/HDL	2.01 ± 0.50	2.12 ± 0.61	2.14 ± 0.59	0.46	0.48
CHOL/HDL	4.46 ± 2.57	4.03 ± 1.04	4.21 ± 1.31	0.32	0.39
**Inflammatory marker**					
hs-CRP (mg/ L)	5.50 ± 5.39	3.78 ± 4.75	4.19 ± 4.13	0.14	0.21

Variables are presented as mean ± SD for continuous

*P* values from t test for continuous variables

Significant items with a *P* value < 0.05 are bolded

*PA* physical activity, *BMI* Body mass index, *WC* waist circumference, *WHR* waist height ratio, *BFM* body fat mas, *FFM* fat free mass, *VFL* visceral fat level, *SBP* Systolic blood pressure, *DBP* Diastolic Blood Pressure, *FBS* Fasting blood sugar, *HOMA-IR* Homeostatic Model Assessment for Insulin Resistance, *TG* Triglyceride, *LDL* Low density lipoprotein, *HDL*, High density lipoprotein, *hs-CRP* High sensitivity C-reactive protein

^*^P-value was found by ANCOVA, and adjusted for age, BMI, physical activity, and total energy intake

^a^BMI considered as collinear, and adjusted for age, physical activity, and total energy intake

^b^Removed the collinear variable from the GLM as confounders

### Dietary intake of study population among MND tertiles

Dietary intakes of the individuals across MND tertiles are shown in Table 2. The results of the comparison after adjusting for energy intake showed that mean protein, fat, MUFA, linoleic acid, EPA, DHA, potassium, vitamin A, C, K, B3, B6, B9, B5, biotin, phosphorus, potassium, zinc, copper, magnesium, selenium, and all components of MND food groups were statistically different (*P* < 0.001).

**Table 2:** Dietary intake of study population among MND tertiles

	**T1 (*n* = 73)**	**T2 (*n* = 160)**	**T3 (*n* = 158)**	*P*-value*	*P*-value**
**Macronutrients**					
Energy intake(kcal)	2080.39 ± 632.61	2514.71 ± 767.23	3008.79 ± 741.26	** < 0.001**	**-**
Protein (g/d)	67.68 ± 21.36	84.65 ± 27.09	108.96 ± 29.79	** < 0.001**	** < 0.001**
Carbohydrate (g/d)	289.23 ± 96.02	351.16 ± 113.78	432.45 ± 117.97	** < 0.001**	0.06
Fat (g/d)	77.17 ± 29.25	93.54 ± 36.86	105.04 ± 32.44	** < 0.001**	**0.004**
**Fat subgroups**					
Cholesterol (mg/d)	215.65 ± 103.32	254.54 ± 110.55	296.07 ± 110.92	** < 0.001**	0.84
SFA (mg/d)	23.36 ± 9.88	27.45 ± 11.41	31.70 ± 11.43	** < 0.001**	0.06
MUFA (mg/d)	25.91 ± 11.38	31.86 ± 13.70	34.97 ± 11.78	**0.001**	**0.015**
PUFA (mg/d)	16.42 ± 9.19	20.21 ± 10.16	21.63 ± 8.68	** < 0.001**	0.10
Linoleic (g/d)	14.48 ± 8.67	17.76 ± 9.57	18.37 ± 8.27	**0.007**	**0.020**
Linolenic (g/d)	1.00 ± 0.59	1.13 ± 0.69	1.39 ± 0.62	** < 0.001**	0.50
EPA (g/d)	0.01 ± 0.03	0.02 ± 0.02	0.04 ± 0.04	** < 0.001**	** < 0.001**
DHA (g/d)	0.04 ± 0.10	0.07 ± 0.07	0.14 ± 0.13	** < 0.001**	** < 0.001**
Trans fatty acids (g/d)	0.00 ± 0.00	0.00 ± 0.00	0.00 ± 0.00	0.25	0.40
**Vitamins**					
Vitamin A (RAE-mcg/d)	486.22 ± 28.38	647.59 ± 281.32	1007.62 ± 428.20	** < 0.001**	** < 0.001**
Vitamin D (μg/d)	1.49 ± 1.38	1.75 ± 1.27	2.38 ± 1.78	** < 0.001**	0.08
Vitamin E (mg/L)	13.66 ± 8.49	17.55 ± 9.88	18.01 ± 8.05	** < 0.001**	0.10
Vitamin k (mcg/d)	177.30 ± 177.38	258.47 ± 270.48	366.74 ± 332.56	** < 0.001**	** < 0.001**
Vitamin B1 (mg/d)	1.76 ± 0.57	2.03 ± 0.73	2.42 ± 0.69	** < 0.001**	0.29
Vitamin B2 (mg/d)	1.81 ± 0.73	2.10 ± 0.80	2.66 ± 0.83	** < 0.001**	0.06
Vitamin B3 (mg/d)	19.67 ± 6.75	24.64 ± 8.69	31.18 ± 10.42	** < 0.001**	**0.007**
Vitamin B5 (mg/d)	4.86 ± 1.87	5.87 ± 1.76	7.76 ± 2.47	** < 0.001**	** < 0.001**
Vitamin B6 (mg/d)	1.57 ± 0.44	1.99 ± 0.62	2.69 ± 0.68	** < 0.001**	** < 0.001**
Biotin (mg/d)	25.71 ± 12.54	34.29 ± 11.89	48.07 ± 17.35	** < 0.001**	** < 0.001**
Vitamin B9 (μg/d)	493.29 ± 147.10	580.66 ± 183.01	719.04 ± 172.36	** < 0.001**	** < 0.001**
Vitamin B12 (mg/d)	3.61 ± 2.70	3.97 ± 1.99	5.06 ± 2.64	** < 0.001**	0.27
Vitamin C (mg/d)	123.25 ± 82.52	163.25 ± 112.56	243.98 ± 110.39	** < 0.001**	** < 0.001**
**Minerals**					
Sodium (mg/d)	3691.58 ± 1746.51	4412.66 ± 1657.03	4919.27 ± 1734.17	** < 0.001**	0.40
Potassium (mg/d)	3056.18 ± 1075.04	4066.22 ± 1398.84	5630.65 ± 1565.56	** < 0.001**	** < 0.001**
Magnesium (mg/d)	329.84 ± 106.28	441.70 ± 140.36	577.46 ± 162.80	** < 0.001**	** < 0.001**
Calcium (mg/d)	1023.96 ± 438.88	1164.02 ± 486.77	1148.57 ± 542.75	** < 0.001**	0.12
Manganese (mg/d)	5.84 ± 2.41	7.93 ± 3.88	9.19 ± 4.40	** < 0.001**	0.14
Zinc (mg/d)	9.92 ± 3.41	12.47 ± 4.25	15.97 ± 4.71	** < 0.001**	** < 0.001**
Iron (mg/d)	19.85 ± 13.96	25.77 ± 21.35	30.13 ± 22.36	**0.002**	0.73
Phosphorus (mg/d)	1259.41 ± 428.15	1543.73 ± 470.75	1999.69 ± 525.34	** < 0.001**	** < 0.001**
Selenium (μg/d)	97.04 ± 33.16	123.71 ± 46.05	142.86 ± 52.74	** < 0.001**	** < 0.001**
Copper (mg/d)	1.47 ± 0.53	1.84 ± 0.58	2.45 ± 0.74	** < 0.001**	** < 0.001**
**MND components**					
Fish (g/d)	5.89 ± 10.36	9.13 ± 8.31	15.15 ± 14.51	** < 0.001**	** < 0.001**
Root vegetables (g/d)	45.21 ± 22.20	66.78 ± 40.66	118.46 ± 58.89	** < 0.001**	** < 0.001**
Apples, pears/high antioxidant fruits (g/d)	63.30 ± 44.32	110.09 ± 84.17	179.31 ± 117.23	** < 0.001**	** < 0.001**
Cabbages (g/d)	147.83 ± 97.48	251.56 ± 205.44	412.25 ± 199.80	** < 0.001**	** < 0.001**
Oatmeal (g/d)	28.62 ± 15.61	45.15 ± 34.36	77.09 ± 53.50	** < 0.001**	** < 0.001**
Rye/ wholegrain breads (g/d)	22.51 ± 25.68	62.68 ± 69.10	77.78 ± 88.40	** < 0.001**	** < 0.001**

*SFA* saturated fatty acid, *MUFA* mono unsaturated fatty acid, *PUFA* poly unsaturated fatty acid, *EPA* Eicosapentaenoic acid, *DHA* Docosahexaenoic acid

Significant items with a *P* value < 0.05 are bolded

^*^P-values from independent samples t test

^**^P -value reported after adjusting energy intake with ANCOVA

### Association between biochemical parameters, body composition and MC4R rs17782313 genotypes

Participants were categorized based on rs17782313 genotypes and divided into two groups: TT genotype (*n* = 152), and TC + CC genotype (*n* = 128) (Table 3). After adjustment for confounding factors (BMI, age, total energy intake, and PA), we found significant differences between genotypes for height (*P* = 0.03) (Table 3).

**Table 3:** Characteristics of the study participants among MC4R genotypes

Variable	TT (*n* = 152)	TC + CC (*n* = 128)	*P*-value	*P*-value*
**Demographic variables**				
Age (years)	37.23 ± 8.65	35.77 ± 8.03	0.14	0.15^b^
PA (MET-minutes/week)	1238.64 ± 1892.47	1177.10 ± 2371.62	0.82	0.96^b^
**Anthropometric parameters**				
Weight (kg)	81.06 ± 12.32	80.28 ± 12.48	0.60	0.44^a^
Height (m)	161.90 ± 5.85	160.98 ± 5.81	0.19	**0.03**^**a**^
BMI (kg/m^2^)	31.06 ± 4.66	30.78 ± 3.90	0.58	0.86^b^
WC (cm)	99.16 ± 10.31	98.53 ± 9.81	0.60	0.63^a^
WHR	0.93 ± 0.05	1.64 ± 8.04	0.27	0.95^a^
**Body composition**				
BFM (kg)	34.02 ± 8.99	33.68 ± 8.30	0.74	0.64^a^
FFM (kg)	47.27 ± 5.53	46.23 ± 5.57	0.11	0.09^a^
VFL	16.42 ± 11.41	16.93 ± 17.32	0.76	0.44^a^
**Blood pressure**				
SBP (mmHg)	112.16 ± 14.04	110.92 ± 13.78	0.46	0.49
DBP (mmHg)	77.92 ± 9.59	77.49 ± 9.87	0.71	0.71
**Blood parameters**				
FBS (mmol/dl)	87.94 ± 10.21	86.24 ± 8.46	0.16	0.63
HOMA-IR	3.36 ± 1.36	3.27 ± 1.17	0.61	0.46
Total Cholesterol (mg/dl)	185.19 ± 34.12	184.83 ± 38.08	0.93	0.63
TG (g/dl)	127.89 ± 74.23	114.52 ± 64.32	0.13	0.30
HDL (mg/dl)	46.35 ± 9.94	47.07 ± 11.98	0.60	0.46
LDL (mg/dl)	95.81 ± 23.23	93.84 ± 25.24	0.52	0.56
LDL/HDL	2.13 ± 0.58	2.06 ± 0.60	0.39	0.31
CHOL/HDL	4.17 ± 1.23	4.18 ± 1.83	0.95	0.78
**Inflammatory marker**				
hs-CRP (mg/ L)	4.15 ± 4.53	4.56 ± 4.77	0.50	0.69

Variables are presented as mean ± SD for continuous variables

*P* values from t test for continuous variables

*PA* physical activity, *BMI* Body mass index, *WC* waist circumference, *WHR* waist height ratio, *BFM* body fat mas, *FFM* fat free mass, *VFL* visceral fat level, *SBP* Systolic blood pressure, *DBP* Diastolic Blood Pressure, *FBS* Fasting blood sugar, *HOMA-IR* Homeostatic Model Assessment for Insulin Resistance, *TG* Triglyceride, *LDL* Low density lipoprotein, *HDL*, High density lipoprotein, *hs-CRP* High sensitivity C-reactive protein

^*^P-value was found by ANCOVA, and adjusted for age, BMI, physical activity, and total energy intake

^a^BMI considered as collinear, and adjusted for age, physical activity, and total energy intake

^b^Removed the collinear variable from the GLM as confounders

### Interactions between MND and MC4R rs17782313 genotypes on cardiovascular risk factors, anthropometric measurements, and body composition

**Fig. 1:**
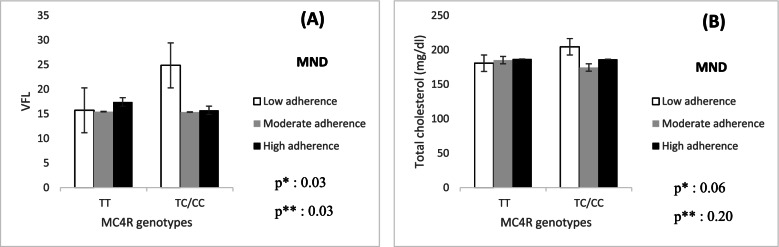
The interaction between MC4R genotypes and MND scores on; (**a**) VFL and (**b**) total cholesterol. *P-value for curd model. **P-value for the adjusted model by energy intake, age, and DBP

Using the GLZM, the interaction between MC4R polymorphism (rs17782313) and MND score (tertiles) on CVD was examined. For this analysis, the TT genotype and low intake were considered as reference groups. A significant negative interaction was observed between the moderate MND score and rs17782313 genotypes on levels of VFL (*P* = 0.07), total cholesterol (*P* = 0.01), LDL (*P* = 0.01), LDL/HDL (*P* = 0.06), Chol/HDL (*P* = 0.06), and hs-CRP (*P* = 0.07) in the crude model. After adjusting for BMI, PA, energy intake, age, family history of obesity, and occupation, all the interactions remained significant. In addition, significant negative interactions were observed between the high MND score and rs17782313 SNP in terms of VFL (*P* = 0.03) and total cholesterol (*P* = 0.06) in the crude model. After adjusting confounders, the interaction between high MND scores and VFL remained significant (Table 4). Generally, we observed that A-allele carriers who had higher MND scores, had lower levels of VFL, total cholesterol, and Chol/HDL, as compared to GG homozygotes (Fig. 1).

**Table 4:** The interaction of MC4R genotypes and MND on CVD risk factors*

Variables	models	alleles	T2			T3		
			β	95% CI	***P***-value	β	95% CI	***P***-value
BMI (kg/m^2^)	Crude	TT		References	0.70		References	0.93
		TC + CC	0.58	-2.46 to 3.63		0.12	-2.85 to 3.10	
	Adjusted	TT		References	0.71		References	0.94
		TC + CC	0.50	-2.18 to 3.20		-0.08	-2.71 to 2.54	
WC (cm)	Crude	TT		References	0.58		References	0.70
		TC + CC	1.95	-5.13 to 9.04		1.34	-5.58 to 8.26	
	Adjusted	TT		References	0.57		References	0.77
		TC + CC	1.94	-4.85 to 8.74		0.97	-5.66 to 7.62	
WHR	Crude	TT		References	0.36		References	0.99
		TC + CC	1.75	-2.05 to 5.56		0.01	-3.70 to 3.73	
	Adjusted	TT		References	0.39		References	0.85
		TC + CC	1.87	-2.41 to 6.16		-0.40	-4.58 to 3.78	
BFM (kg)	Crude	TT		References	0.42		References	0.54
		TC + CC	2.50	-3.60 to 8.60		1.86	-4.10 to 7.82	
	Adjusted	TT		References	0.49		References	0.79
		TC + CC	1.99	-3.66 to 7.66		0.73	-4.79 to 6.26	
FFM (kg)	Crude	TT		References	0.98		References	0.40
		TC + CC	0.03	-3.84 to 3.90		-1.62	-5.40 to 2.16	
	Adjusted	TT		References			References	0.32
		TC + CC	-0.21	-4.19 to 3.75	0.91	-1.95	-5.84 to 1.92	
VFL	Crude	TT		References	**0.07**		References	**0.03**
		TC + CC	-9.23	-19.25 to 0.79		-10.84	-20.64 to -1.04	
	Adjusted	TT		References	**0.07**		References	**0.03**
		TC + CC	-10.25	-21.53 to 1.02		-12.22	-23.24 to -1.20	
HOMA-IR	Crude	TT		References	0.54		References	0.87
		TC + CC	0.29	-0.65 to 1.23		-0.07	-1.06 to 0.90	
	Adjusted	TT		References	0.70		References	0.82
		TC + CC	0.19	-0.81 to 1.21		0.11	-0.93 to 1.17	
Total Cholesterol (mg/dl)	Crude	TT		References	**0.01**		References	**0.06**
		TC + CC	-34.64	-61.30 to -7.97		-24.24	-49.87 to 1.38	
	Adjusted	TT		References	**< 0.001**		References	0.20
		TC + CC	-38.79	-65.00 to -12.58		-16.29	-41.35 to 8.77	
TG (g/dl)	Crude	TT		References	0.62		References	0.79
		TC + CC	-13.17	-65.80 to 39.44		-6.70	-57.28 to 43.87	
	Adjusted	TT		References	0.61		References	0.72
		TC + CC	-14.31	-70.39 to 41.76		9.51	-44.11 to 63.15	
HDL (mg/dl)	Crude	TT		References	0.75		References	0.46
		TC + CC	-1.30	-9.53 to 6.92		-2.94	-10.85 to 4.96	
	Adjusted	TT		References	0.77		References	0.72
		TC + CC	1.23	-7.25 to 9.71		-1.44	-9.56 to 6.67	
LDL (mg/dl)	Crude	TT		References	**0.01**		References	0.17
		TC + CC	-21.69	-39.71 to -3.67		-11.90	-29.22 to 5.41	
	Adjusted	TT		References	**0.03**		References	0.53
		TC + CC	-18.77	-36.59 to -0.95		-5.41	-22.45 to 11.62	
LDL/HDL	Crude	TT		References	**0.06**		References	0.52
		TC + CC	-0.08	-0.18 to 0.00		-0.02	-0.11 to 0.06	
	Adjusted	TT		References	**0.04**		References	0.70
		TC + CC	-0.96	-0.19 to -0.00		-0.01	-0.10 to 0.07	
CHOL/HDL	Crude	TT		References	**0.06**		References	0.20
		TC + CC	-1.08	-1.81 to 0.39		-0.70	-1.81 to 0.39	
	Adjusted	TT		References	**0.01**		References	0.19
		TC + CC	-1.50	-2.23 to 0.05		-0.76	-1.91 to 0.39	
hs-CRP (mg/ L)	Crude	TT		References	**0.07**		References	0.34
		TC + CC	-3.24	-6.76 to 0.26		-1.63	-4.99 to 1.72	
	Adjusted	TT		References	0.12		References	0.51
		TC + CC	-2.90	-6.62 to 0.80		-1.17	-4.72 to 2.37	

TT genotype has 0 risk allele. TC genotype has one and CC genotype have two risk alleles

TT genotype and Low adherence to MND are considered as references

Generalized linear model; crude model and adjusted model to BMI, physical activity, kcal, age, family history of obesity, and occupation, as covariates

*BMI* Body mass index, *WC* waist circumference, *WHR* waist height ratio, *BFM* body fat mas, *FFM* fat free mass, *VFL* visceral fat level, *TG* Triglyceride, *LDL* Low density lipoprotein, *HDL*, High density lipoprotein, *CHOL* cholesterol, *hs-CRP* High sensitivity C-reactive protein

^*^For interactions, *P* < 0.1 was considered significant

## Discussion

The current cross-sectional study was conducted to investigate the interaction between the MND and MC4R gene variants on CVD risk factors in overweight and obese Iranian women. Our results showed a negative interaction between MND scores and MC4R gene variants on cholesterol and VFL levels. In line with our study, a cross-sectional study in 2020 evaluated the interaction between dietary inflammatory index (DII) and MC4R gene variants on cardiovascular risk factors and found an interaction between DII score and rs17782313 polymorphism on total cholesterol and body composition (soft lean mass (SLM), fat-free mass (FFM), skeletal muscle mass (SMM)). The authors suggested that dietary compositions, gene variants, and their interaction, should be considered in CVD risk assessment [54].

The effects of MC4R genetic variants on body composition, as well as some metabolic parameters, may depend on dietary factors. Our findings highlight a gene-diet interaction for moderate adherence to MND and MC4R polymorphism (rs17782313) on cholesterol, LDL, LDL/HDL, and Chol/HDL levels in both crude and adjusted models. The reason why we observed such an interaction is plausibly attributable to the baseline metabolic levels between the two groups not being significantly different, so the actual interaction between the two groups was not detectable. Ramezani-Jolfaie et al., reported that the ND can improve BP, and also some blood lipid markers (total cholesterol and LDL), and it should be considered as a healthy dietary pattern [61]. A randomized controlled trial, that included 88 hypercholesterolaemic subjects, showed that ND decreased cholesterol, LDL, and LDL/HDL levels compared to the control group [62]. Many environmental and individual characteristics can affect gene-diet interaction, thus, another reason that we were not able to see the interaction between high adherence to MND and MC4R is conceivably due to that all individuals in our study were healthy; perhaps if we recruited participants with pre-existing metabolic syndrome, different interaction results would be evident. Therefore, future studies are warranted to further understand this interaction on CVD risk factors, particularly in those with pre-existing co-morbidities.

In the current study, we also observed an interaction between moderate adherence to MND and MC4R on hs-CRP; however, this interaction was attenuated in the adjusted model and high adherence to this diet. A narrative review of two observational and eight intervention studies reported an inverse association between high adherence to the ND pattern and concentration of hs-CRP [63]. The ND is a plant-based diet, including legumes, whole grain cereals, and dietary fiber from oats and barley [64]. It is well known that replacing saturated fat with polyunsaturated fat reduces LDL [65]. It has also been suggested that replacing saturated fat with polyunsaturated or monounsaturated fat improves insulin sensitivity and other metabolic parameters [66]. Furthermore, the increase in dietary fiber from whole grains, legumes, fruits, and vegetables may have beneficial health effects. Additionally, as we mentioned in the introduction section, ND contains berries and fruits which are major sources of antioxidants, hence, as dietary antioxidants, they might inhibit adiposity by regulating brown adipose tissue metabolism, augmenting thermogenesis, and reducing adiponectin and leptin gene expression in adipocytes [67, 68].

The main strength of the present study is that although several studies have assessed the interaction of MC4R and diet on metabolic syndrome, diabetes, and obesity [69, 70], no investigations have been conducted to assess the interaction between MND and MC4R rs17782313 polymorphism on CVD risk factors.

Although we provide novel findings of gene-diet interactions, some limitations should be considered in the interpretation of the study. The results of this cross-sectional study, although nationally illustrative, cannot indicate a causal relationship. In addition, even though we applied a validated FFQ, measurement errors (such as recall bias) [71] are possible. Moreover, our study only included women, thus, the results are not generalizable to men. Although participants were randomly selected from 4 different areas of Tehran (North, South, East, and West), they were volunteers, so were more likely to be healthier and may not be representative of the general population of women in Iran. Also, it was not possible to determine the exact mechanism of the relationship between MND and the rs17782313 genotype because all the subjects were healthy, and baseline parameters were not significantly different between MND tertiles. In the present study, only the most common SNP in the MC4R gene was examined, and other SNPs are suggested to be investigated by future studies. Finally, although we considered potential confounders, residual confounding may still exist.

## Conclusion

In conclusion, our results showed significant negative interactions between high MND score and rs17782313 SNP in terms of VFL and total cholesterol in the crude model. After adjusting confounders, the interaction between high MND score and VFL remained significant. However, there were no interactions between MND and MC4R rs17782313 polymorphism on cardiovascular risk factors. The present study recommends that diet, gene variants, and their interaction should be considered in metabolic diseases risk assessment, and further studies are needed and should consist of diverse ages and genders, considering clinical vs. non-clinical populations, and larger sample sizes, to confirm the veracity of these data and better elucidate the interaction.

## Supplementary Information

**Additional file 1: Supplementary table 1.** Normal ranges of anthropometric measurements, blood pressure and blood parameters.

## Data Availability

The data that support the findings of this study are available from Khadijeh Mirzaei but restrictions apply to the availability of these data, which were used under license for the current study, and so are not publicly available. Data are however available from the authors upon reasonable request and with permission of Khadijeh Mirzaei.

## Abbreviations

CVD: Cardiovascular disease
DASH: Dietary approaches to stop hypertension
MDS: Mediterranean diet score
HEI: Healthy eating index
ND: Nordic diet
MC4R: Melanocortin 4 receptor
BP: Blood pressure
BMI: Body mass index
PCOS: Polycystic ovary syndrome
NAFLD: Nonalcoholic fatty liver disease
HTN: Hypertension
FFQ: Food frequency questionnaire
PCR: Polymerase chain reaction
WC: Waist circumference
BFM: Body fat mass
FFM: Fat-free mass
VFL: Visceral fat level
BIA: Bioelectrical impedance analysis
TG: Triglyceride
HDL: High-density lipoprotein cholesterol
LDL: Low-density lipoprotein
hs-CRP: High sensitivity C-reactive protein
IPAC: International Physical Activity Questionnaire
ANOVA: Analysis of variance
ANCOVA: Analysis of covariance
MND: Modified Nordic diet
SD: Standard deviation
EPA: Eicosapentaenoic acid
DHA: Docosahexaenoic acid
GLZM: Generalized linear regression model
SLM: Soft Lean Mass
SMM: Skeletal Muscle Mass
SNP: Single nucleotide polymorphism
