# Supplementary material for: Are there any interactions between modified Nordic-style diet score and MC4R polymorphism on cardiovascular risk factors among overweight and obese women? A cross-sectional study
Source: BMC Endocr Disord. 2022 Sep 1;22:221. doi: 10.1186/s12902-022-01132-1 (PMC9434967; doi:10.1186/s12902-022-01132-1)
Supplement: Supplementary file 1 — Additional file 1: Supplementary table 1. Normal ranges of anthropometric measurements, blood pressure and blood parameters. [file 12902_2022_1132_MOESM1_ESM.docx]

| **Supplementary table 1. Normal ranges of anthropometric measurements, blood pressure and blood parameters.** | |
| --- | --- |
| **parameters** | **Normal ranges** |
| **Anthropometric measurements** | |
| BMI | 18.5-24.9 kg/m^2^ |
| WC | ≤88 cm in women |
| WHR | ≤0.85 in women |
| **Blood pressure** | |
| SBP | Less than 120 mmHg |
| DBP | Less than 80 mmHg |
| **Blood parameters** | |
| FBS | Less than 100 mg/dl |
| HOMA-IR | Less than 1.77 (for Iranian population) |
| Total cholesterol | Less than 200 mg/dl |
| TG | Less than 150 mg/dl |
| HDL | Above 40 mg/dl |
| LDL | Less than 130 mg/dl |
| hs-CRP | Less than 3.00 mg/l |
